# The Pretext

**Published:** 1896-07

**Authors:** 


THE PRETEXT.—As desirable as it is that the confiding, and too often ignorant and credulous, should be protected from the dangers of the doctor, for it is not alone the ignorant practitioner who constitutes the danger, but the well educated fraud, I contend that the State Medical Association, representing as it does as yet only about ten per cent. of the profession of the State, has no authority to assume the guardianship of the public health, and are not responsible for it. They have done their duty in this connection as they conceived it, and have earnestly sought to get legislative protection thrown around the public health. But it has been refused, and the Association should wash its hands of the matter; and in future, when the people see and realize the danger we have in vain sought to point out and guard against, they will petition for the protection so often refused at the request of the Association.

It is urged that the step now proposed to be taken is demanded by the situation, that it is done as a matter of "expediency." I contend that the emergency does not exist; the responsibility of the regular profession for the public safety is not so great as to justify the sacrifice the State Association proposes to make to compass it; and being "compassed," the end and object are only partially attained; the people are not protected; but left exposed to the dangers of a large number of the most dangerous element, the alleged homeopaths. If the people are to be "protected," let them be protected from all quacks; do not let us take the biggest half of the biggest element of quacks into co-partnership with us in the protecting business

